# Rate and Predictors of Unforeseen PN1/PN2-Disease in Surgically Treated cN0 NSCLC-Patients with Primary Tumor > 3 cm: Nationwide Results from Italian VATS-Group Database

**DOI:** 10.3390/jcm12062345

**Published:** 2023-03-17

**Authors:** Filippo Lococo, Dania Nachira, Marco Chiappetta, Isabella Sperduti, Maria Teresa Congedo, Elisa Meacci, Fausto Leoncini, Rocco Trisolini, Roberto Crisci, Carlo Curcio, Monica Casiraghi, Stefano Margaritora, on the behalf of the Italian VATS Group

**Affiliations:** 1 Università Cattolica del Sacro Cuore, 00168 Rome, Italy; 2 Thoracic Surgery, Fondazione Policlinico Universitario A. Gemelli IRCCS, 00168 Rome, Italy; 3 Biostatistics, IRCCS Regina Elena National Cancer Institute, 00144 Rome, Italy; 4 Interventional Pulmonology Unit, Fondazione Policlinico Universitario A. Gemelli IRCCS, 00168 Rome, Italy; 5 Thoracic Surgery, University of L’Aquila, 67100 L’Aquila, Italy; 6 Division of Thoracic Surgery, Monaldi Hospital, 80100 Naples, Italy; 7 Department of Thoracic Surgery, IEO-European Institute of Oncology IRCCS, 20141 Milan, Italy; 8 Department of Oncology and Hemato-Oncology, University of Milan, 20141 Milan, Italy

**Keywords:** NSCLC, surgery, nodal upstaging, staging, VATS, VATS-Group

## Abstract

**Background.** Since no robust data are available on the real rate of unforeseen N1-N2 disease (uN) and the relative predictive factors in clinical-N0 NSCLC with peripheral tumours > 3 cm, the usefulness of performing a (mini)invasive mediastinal staging in this setting is debated. Herein, we investigated these issues in a nationwide database. **Methods.** From 01/2014 to 06/2020, 15,784 thoracoscopic major lung resections were prospectively recorded in the “Italian VATS-Group” database. Among them, 1982 clinical-N0 peripheral solid-type NSCLC > 3 cm were identified, and information was retrospectively reviewed. A mean comparison of more than two groups was made by ANOVA (Bonferroni correction for multiple comparisons), while associations between the categorical variables were estimated with a Chi-square test. The multivariate logistic regression model and Kaplan–Meyer method were used to identify the independent predictors of nodal upstaging and survival results, respectively. **Results.** At pathological staging, 229 patients had N1-involvement (11.6%), and 169 had uN2 disease (8.5%). Independent predictors of uN1 were SUVmax (OR: 1.98; CI 95: 1.44–2.73, *p* = 0.0001) and tumour-size (OR: 1.52; CI: 1.11–2.10, *p* = 0.01), while independent predictors of uN2 were age (OR: 0.98; CI 95: 0.96–0.99, *p* = 0.039), histology (OR: 0.48; CI 95: 0.30–0.78, *p* = 0.003), SUVmax (OR: 2.07; CI 95: 1.15–3.72, *p* = 0.015), and the number of resected lymph nodes (OR: 1.03; CI 95: 1.01–1.05, *p* = 0.002). **Conclusions.** The unforeseen N1-N2 disease in cN0/NSCLCs > 3 cm undergoing VATS resection is observable in between 12 and 8% of all cases. We have identified predictors that could guide physicians in selecting the best candidate for (mini)invasive mediastinal staging.

## 1. Introduction

The treatment plan in non-small cell lung cancer (NSCLC) is substantially based on the tumour stage and general clinical condition of the patient. Basically, while surgery remains the best treatment in clinical N0/N1 disease, multimodal combined treatment (including surgery) is preferable in N2 disease. Therefore, the clinical stage (performed by imaging and (mini)invasive procedures) should be as accurate as possible in order to reduce, at minimum, the number of unforeseen node diseases (pN1-N2) at pathological staging after surgery [1].

Indeed, pathological nodal involvement is one of the most important prognostic factors in NSCLC to evaluate any possible adjuvant therapy and is a good parameter for the effectiveness of the lymphadenectomy and, therefore, of the surgical approach employed [2].

Since no robust data are available on the real rate of unforeseen N1-N2 disease (uN), its prognostic impact, and the relative predictive factors in clinical-N0 NSCLC with peripheral tumours > 3 cm, means that the usefulness of performing a (mini)invasive mediastinal staging in this setting is debated. 

In particular, the role of (mini)invasive mediastinal procedures for patients with no detectable lymph node metastases on imaging studies is unclear, and it is questionable whether aggressive invasive lymph node staging affects the prognosis of patients with clinical stage I disease on imaging.

The primary endpoint of this work is to investigate the rate and (secondary endpoint) predictors of unforeseen pN1/pN2-disease in surgically treated cN0-NSCLC patients with a primary tumour > 3 cm in a nationwide database.

## 2. Materials and Methods

### 2.1. Patients

All the data used in this analysis were retrospectively extracted from the Italian VATS Group Registry. This database was created in January 2014 to prospectively collect data on VATS lobectomies performed by 56 Italian-certified thoracic surgery centres. Among 15784 cases, 1982 met the inclusion criteria for our study (diagnosis of NSCLC, clinical N0, solid-type tumour measuring > 3 cm).

**Figure 1:**
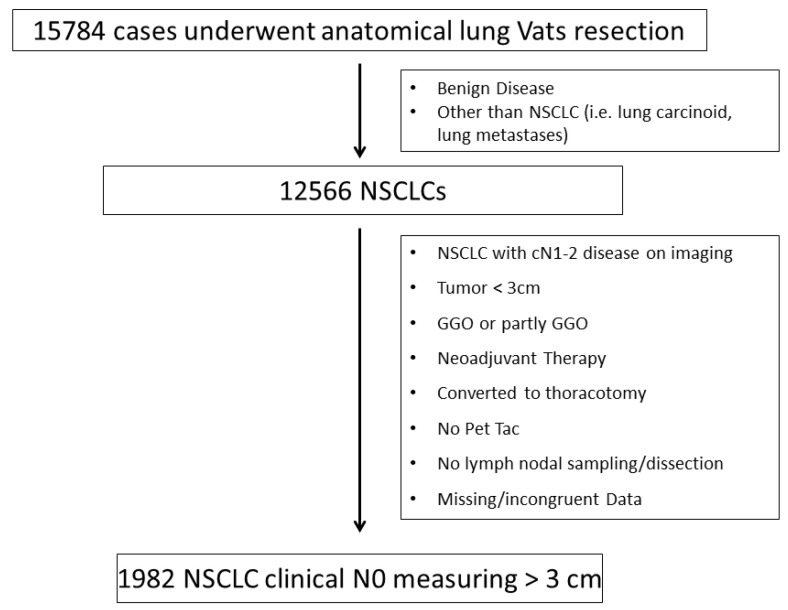
Consort diagram.

We excluded from our population study those patients who did not undergo a PET/CT scan before surgery and those with suspected Hilo-mediastinal lymph nodes at CT and/or PET, even if investigated by the (mini) invasive staging of the mediastinum [3]. Moreover, we excluded NSCLC patients who were converted to thoracotomy, those who underwent neoadjuvant treatments, and those with a pathological diagnosis that was different from NSCLC (see the consort diagram reported in Figure 1). We also did not consider patients with sub-solid/non-solid NSCLC, synchronous lung cancer, or multiple nodules.

For each patient, we recorded the preoperative characteristics such as the age, sex, clinical (c) TNM, intraoperative details, pathological (p) TNM, and the final pathology report, excluding cases where these variables were incomplete or data incongruent.

Despite unavoidable differences between centres, the principles at the basis of surgical lymph node dissection were those described by ESTS [4]. As well, the definitions of systematic nodal dissection, systematic nodal sampling, and nodal sampling followed the ESTS standardized tassonomy defined in 2004 [4].

The rate of nodal upstaging was defined by comparing cTNM to pTNM based on the eighth edition of TNM classification [5]. Nodal micrometastases are defined as clusters of cells measuring between 0.2 and 2 mm in their greatest diameter, usually with mitoses and vascular or lymphatic invasion, and when identified, are considered similar to other nodal metastases with consequent tumour upstaging [6].

### 2.2. Statistical Methods

Descriptive statistics were used to summarize the pertinent study information. Associations between categorical variables were analysed according to the Pearson chi-square test or Fisher exact test when indicated.

Survival curves were calculated by the Kaplan–Meier method from the date of surgery until relapse or death. The log-rank test was used to assess differences between the subgroups. Significance was defined at the *p* ≤ 0.05 level.

The odds ratio (OR) and the 95% confidence intervals (95% CI) were estimated using the logistic regression model. 

Factors considered for the univariable analysis of uN1 and uN2 occurrence were: age (continuous), sex, side, type of surgery, histology, lymphadenectomy, primary tumour SUVmax, tumour size, tumour location, the number of resected nodes, type of surgical approach. 

Multivariate logistic regression was developed using stepwise regression (forward selection, enter with a limit or a removed limit, *p* = 0.10 and *p* = 0.15, respectively) to identify independent predictors of outcome.

The SPSS (version 21.0; SPSS, Inc., Chicago, IL, USA) and MedCalc (version 14.2.1; MedCalc Software, Ostend, Belgium) licensed statistical programs were used for all analyses.

All of the patients included in this national registry gave their written informed consent, and this database project was approved by the Institutional Research Review Board (IRRB) of each participating centre.

## 3. Results

From 01/2014 to 06/2020, 15784 thoracoscopic major lung resections were prospectively recorded in the “Italian VATS-Group” database. Among them, the data of 1982 patients who underwent VATS lobectomy from

January 2014 to April 2017 and met the inclusion criteria were extracted from the database (Figure 1) and retrospectively analysed. 

Among those, 1315 (67%) were males, 667 (33%) were females, and the median age was 69.7 ± 8.9 years. The main clinical and surgical features are summarized in Table 1.

In particular, the tumour size distribution was: 3–5 cm in 76.9% of cases, 5–7 cm in 18.8%, and >7 cm in 4.1%. The main histology was adenocarcinoma (more than 70% of all tumours), while the median uptake SUVmax value was 8.7 ± 6.4. The surgery consisted of an anatomical resection, in all cases, with only 1.6% of sublobar resection. The main surgical approach was triportal (78%; mainly anterior by “Copenhagen”), followed by biportal (11.9%) and uniportal (10.1%). Two-thirds of the patients underwent systematic lymph nodal dissection, with a mean number of dissected lymph nodes at 13.7 ± 8.3. Finally, no marked differences in terms of the nodal stations and the number of lymph nodes harvested were observed between the different approaches used.

### 3.1. Clinico-Pathological Characteristics in N0, uN1 and uN2 Disease

At pathological staging, 229 patients had N1-involvement (11.6%), and 169 had uN2 disease (8.5%). Among pN1 patients, the main number of positive lymph nodes and the rate of micrometastases were 2.0 ± 1.3 and 17.5% (40 patients), respectively, while in pN2 patients, they were 3.0 ± 1.4 and 17.7% (30 patients), respectively.

The distribution of the p-N status according to clinic-pathological variables is reported in Table 2. 

A significant difference (*p* = 0.001) was observed for the uN1/uN2 rate compared to tumours with a low FDG uptake and high FDG uptake, and in addition, higher SUVmax values were associated in patients with uN1/uN2 disease compared with N0 disease. Similarly, the rate of N2 disease was higher in adenocarcinoma (9.8% vs. 4.6%, *p* = 0.008) compared to squamous cell carcinoma, while the rate of uN1 disease was substantially similar. Interestingly, the larger the tumour size, the higher the rate of uN1 disease was (*p* = 0.003), while the rate of uN2 disease was similar in all subgroups. Finally, the mean number of dissected lymph nodes was significantly (*p* < 0.001) higher in uN1/uN2 disease (both 15.7%) compared to that observed in N0-disease (12.4%).

### 3.2. Predictive Factors for uN1 and uN2

Using a multivariate logistic regression model, independent predictors of uN1 were SUVmax (OR: 1.98; CI95%: 1.44–2.73, *p* = 0.0001), and a tumour-size >5 cm (OR: 1.52; CI95%: 1.11–2.10, *p* = 0.01), while independent predictors of uN2 were age (OR: 0.98; CI95%: 0.96–0.99, *p* = 0.039), adenocarcinoma histology (OR: 0.48; CI95%: 0.30–0.78, *p* = 0.003), SUVmax > 6 (OR: 1.807; CI95%: 1.27–2.58, *p* = 0.001), lymph node resected > 6 (OR: 2.37; CI95%: 1.26–4.45, *p* = 0.007) (Table 3).

### 3.3. Survival Results According to N-Status

**Figure 2:**
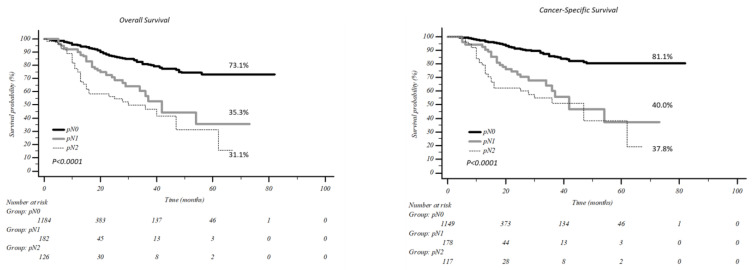
Overall and cancer specific survival according to nodal involvement.

The five-year overall survival (Figure 2) was 73.1% in pN0 patients vs. 35.3% and 31.1% in pN1 and pN2 patients, respectively (*p* < 0.0001), while five-year cancer-specific survival was 81.1% in pN0 vs. 40.0% and 37.8% in pN1 and pN2, respectively (*p* < 0.0001).

**Figure 3:**
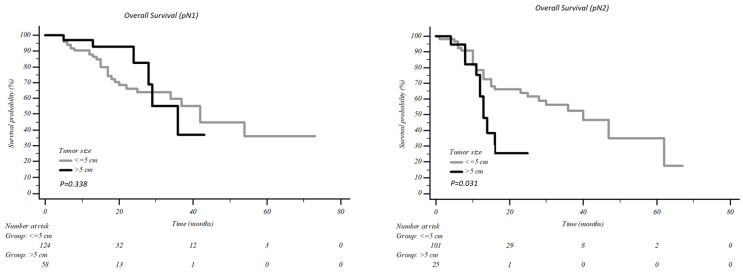
Overall survival according to the tumour dimension in patients with pN1 and pN2.

According to tumour dimension, no difference in survival was present in patients with pN0 and pN1 and a tumour >5 cm vs. <5 cm, 5years overall survival was 74.7% vs. 72.8% (*p* = 0.612) in pN0 patients and 36.0% vs. 36.7% in pN1 patients (*p* = 0.338, Figure 3). Conversely, in uN2 patients, the difference in survival was statistically significant: five-year overall survival of 35.1% was recorded in tumours <5 cm vs. 25.5% in tumours >5 cm (*p* = 0.031, Figure 3). 

## 4. Discussion

The American College of Chest Physicians (ACCP) guidelines [7] and the ESTS guidelines [4] suggest performing mediastinal staging by mediastinoscopy or video-assisted mediastinoscopy (VAM) in some specific subsets of patients with negative lymph nodes at preoperative CT and/or PET-CT. In the case of central tumours, tumours greater than 3 cm or cN1, or with adenocarcinoma histology, the ACCP indicates endoscopic staging by EBUS/EUS with FNA as a first step (level of evidence 2B), while the ESTS concludes that the choice between mediastinoscopy/pre-surgical lymphadenectomy (VAMLA or TEMLA) and EBUS/EUS must rely on local expertise (level of evidence V). The reported sensitivity of EBUS was 0.17–0.41 in cN0 patients and 0.38–0.53 in cN1 ones for early-stage NSCLC, and 0.86–0.88 in N2/N3 NSCLC; the sensitivity of combined EUS/EBUS was 0.83 (95% CI 0.77–0.8). Negative EUS/EBUS results in patients at risk were confirmed by mediastinoscopy, which, to date, remains the gold standard in the staging process, with a sensitivity ranging from 0.78 to 0.97 and a negative predictive value of 0.83–0.99 [3,8]. 

Nevertheless, not all thoracic centres perform mediastinoscopy as a confirmation in patients with suspicious nodal enlargement or large tumours but with negative PET-CT after a negative EBUS [9]. A retrospective study on Italian VATS-Group data concluded that only 3.5% of patients (22.1% cT2 and 1.8% cT3) underwent an invasive mediastinal staging with an incidence of pN2-upstaging of 6.5% [9].

The main risk factors involved in nodal upstaging in early-stage lung cancer are still debated, and several works also investigated the topic in relation to different surgical approaches, including open, VATS, or both. Indeed, while some authors have been concerned about the safety and effectiveness of VATS in performing an oncological radical lymphadenectomy compared to open or RATS surgery, Toker et al. [2] believed that VATS lymphadenectomy could have some limits only in the hands of novice surgeons. In fact, while RATS surgery gives the possibility to everyone to replicate open dissection, thanks to the high technological instrumentations in assisting movements, VATS requires certain expertise. Therefore, after overcoming the learning curve, a surgical approach should not influence nodal upstaging.

In recent years, several—manly retrospective—studies (Table 4) were identified as predictive risk factors for post-operative nodal upstaging: T stage, tumour size, number of dissected nodes, type of surgical approach, lower lobes, SUVmax, adenocarcinoma histology [1,10,11,12,13,14], etc.

In our series of 1982 patients with cN0 peripheral solid-type NSCLC > 3 cm, both the N1- and N-2 involvements were higher (11.6% vs. 6.2% and 8.5% vs. 2.4%) compared to cT1-T3N0 patients of Marulli’s series [13] from the same national database. This could be explained by the presence of only larger tumours (>3 cm) in our dataset. Indeed, a previous retrospective analysis on 160 cN0 NSCLC patients [16] who underwent an open or Uniportal VATS approach identified the main risk factor for pN1 upstaging only in central/larger (>3 cm) tumours (*p*: 0.0004). 

Our analysis confirmed most of the results of previous studies [13,14], identifying, in particular, SUVmax > 6 (*p* = 0.0001) and the tumour size >5 cm (*p* = 0.01) as predictor factors for pN1 nodal upstaging, while age (*p* = 0.039), adenocarcinoma histology (*p* = 0.003), SUVmax > 6 (*p* = 0.001), and more than six lymph nodes resected (*p* = 0.007) as predictors for pN2. In particular, we confirmed the role of histology in predicting N2 upstaging, as other authors reported previously [12,13,14]. The number of retrieved nodes predictive of pathological upstaging is also an argument of debate among the authors. Ismail and colleagues [15] concluded that the resection of 18 nodes could be the best predictor of general nodal upstaging (13.3%) in a single-centre VATS series of 136 patients and suggested the removal of at least seven nodes from hilar stations and eleven from mediastinal ones to enhance the possibilities of detecting an unforeseen nodal disease.

On the other hand, in our study, tumour dimension resulted as a predictive factor for N1 upstaging and not for N2, but this result could explain considering the kind of N1 involvement. Indeed, despite N2, metastases are only due to lymphatic spreading, and N1 involvement may be related to lymphatic dissemination or direct nodal invasion. Unfortunately, in the database, it was not possible to know if N1 positive nodes were related to direct infiltration, but the rate of these cases may explain this difference considering the tumour dimension on this topic.

Interestingly, age resulted in an independent prognostic factor for N2 upstaging, which is, to our knowledge, the first report of this risk factor. However, even if we found a correlation between age and N2 upstaging, it was hard to understand the possible correlation. One possibility was the presence of more aggressive tumours in younger patients [17], but further studies are needed to validate this hypothesis.

Accurate identification of the predictive factors of upstaging is pivotal for selecting the best candidates for a (mini)invasive mediastinal staging. From a theoretical point of view, we could reserve the staging (mini-invasive) procedures only for cN0-NSCLC patients with a higher risk of N+ disease with several practical implications: (i) to optimize resources and reducing costs; (ii) to avoid complications from unnecessary procedures; (iii) to reduce an interval in surgery. Obviously, these results should be confirmed on the prospective clinical cohort of patients, and clear recommendations should guide physicians in the diagnostic pathway.

Moreover, an accurate definition of N-status before surgery has several potential implications. In particular, a correct staging could help in planning the most appropriate and tailored treatment for patients at risk—not only in terms of adjuvant therapies—but to increase their overall survival. Indeed, limited sub-lobar resections or sampling/limited lobe-specific lymphadenectomy should be avoided in patients with risk factors of nodal upstaging.

While some authors [18,19,20] believe that nodal involvement in the post-operative period and unexpected pN2 disease could worsen survival, Obiols et al. [21] showed a reasonable survival rate (40% at 5-year follow-up vs. 10–30%) of that reported in the above-mentioned studies [18,19]. The authors explained the results by accurate preoperative staging, which reduced the number of uN2 if conducted according to the ESTS guidelines. Furthermore, they concluded that surgery might be reasonable in pN2 patients if complete resection can be achieved.

Our survival results showed a five-year overall survival of 35.3% and 31.1% in pN1 and pN2 patients, respectively, compared to 73.1% in pN0 patients (*p* < 0.0001). From these results, the importance of accurately defining the N-status before surgery clearly emerged, even if pN2 patients should not be excluded a priori from surgery but discussed in a multidisciplinary setting.

Our large series reflects the real scenario adopted in most parts of Italian Thoracic Centres in the preoperative management and staging procedures of this subset of NSCLC patients. The main biases of this work are the retrospective nature of the study on a prospective collected national database, the selection bias (only VATS procedures), and above all, the no uniform adherence to ESTS guidelines by thoracic surgeons in performing pre-operative staging in the case of large (>3 cm) peripheral cN0. Moreover, since we found that the mean number of dissected lymph nodes was significantly higher in uN1/uN2 disease compared to that observed in N0-disease, the rate of uN1/N2 could be underestimated in those patients with a few lymph nodes sampled during surgery. Then, the unforeseen postoperative nodal involvement is a mirror for a series of factors, such as the surgeons’ expertise in VATS, the intrinsic risk factors related to the tumour, and, above all, the incorrect preoperative management of those patients.

## 5. Conclusions

The unforeseen N1-N2 disease in cN0/NSCLCs measuring >3 cm and undergoing VATS resection is observed in between 12 and 8% of all cases, respectively, and seems to have a prognostic impact. Considering the importance of identifying predictive factors in this subpopulation of NSCLC patients, we have herein identified different predictors of unforeseen N1 and uN2 on a large cohort of patients who underwent video-assisted surgery.

These findings should be confirmed in prospective studies and, in the near future, could guide physicians to select the best candidate for (mini)invasive mediastinal staging and to adopt a tailored strategy of care.

## Figures and Tables

**Table 1:** Clinical and pathological characteristics.

Variables	
**Gender**	
*M*	1315 (66.3%)
*F*	667 (33.6%)
**Age of Diagnosis**	
*Mean* ± SD	69.7 ± 8.9
*<70 years*	952 (48.0%)
*≥70 years*	1030 (51.9%)
**Side**	
*Left*	828 (41.7%)
*Right*	1154 (58.2%)
**Tumour Location**	
*Upper*	785 (39.6%)
*Middle*	77 (3.8%)
*Lower*	1120 (56.5%)
**Primary tumour PET SUVmax**	
*Mean* ± SD	8.7 ± 6.4
*SUVmax < 2.5*	267 (13.4%)
*SUVmax* ≥ *2.5*	1563 (78.8%)
**Histology**	
*Adenocarcinoma*	1418 (71.5%)
*Squamous cell carcinoma*	456 (23.0%)
*Others*	108 (5.4%)
**Tumour-size**	
*3–5 cm*	1526 (76.9%)
*5–7 cm*	373 (18.8%)
*>7 cm*	83 (4.1%)
**Surgery**	
*(Bi)Lobar Resection*	1949 (98.3%)
*Sublobar Resection*	33 (1.6 %)
**Type of surgical approach**	
*Triportal*	*1546 (78.0%)*
*Biportal*	*236 (11.9%)*
*Uniportal*	*200 (10.1%)*
**Lymph Node Assessment**	
*Radical Dissection*	1341 (67.6%)
*Systematic Sampling or Sampling*	641 (32.3%)
*Number of LFN dissected (Mean* ± SD)	13.7 ± 8.3
**All**	1982

**Table 2:** The distribution of p-N status according to clinic-pathological variables. In this table we describe the clinico-pathological characteristics in the different groups according to N-status.

Variables	pN0 (n, %)	uN1 (n, %)	uN2 (n, %)
Population	1584 (79.9%)	229 (11.6%)	169 (8.5%)
**Gender**			
*M*	1064 (80.9%)	145 (11.0%)	106 (8.1%)
*F*	520 (78.0%)	84 (12.6%)	63 (9.4%)
**Age of Diagnosis**			
*Mean* ± SD	70.3 ± 5.4	69 ± 8.5	68 ± 11.0
*<70 years*	744 (78.2%)	119 (12.5%)	89 (9.3%)
*≥70 years*	840 (81.5%)	110 (10.7%)	80 (7.8%)
**Side**			
*Left*	642 (77.6%)	109 (13.2%)	77 (9.3%)
*Right*	942 (81.6%)	120 (10.4%)	92 (8.0%)
**Tumour Location**			
*Upper*	610 (77.7%)	103 (13.1%)	72 (9.2%)
*Middle*	63 (81.8%)	7 (9.1%)	7 (9.1%)
*Lower*	1001 (89.4%)	119 (10.6%)	90 (8.0%)
**Primary tumour PET SUVmax**			
*Mean* ± SD	7.8 ± 5.9	10.1 ± 6.1	8.8 ± 5.5
*SUVmax < 2.5*	235 (88.0%	19 (7.1%)	13 (4.9%)
*SUVmax* ≥ *2.5*	1226 (78.5%)	191 (12.2%)	146 (9.3%)
**Histology**			
*Adenocarcinoma*	1109 (78.2%)	170 (11.9%)	139 (9.8%)
*Squamous cell carcinoma*	386 (84.7%)	49 (10.7%)	21 (4.6%)
*Others*	89 (82.4%)	10 (9.3%)	9 (8.3%)
**Tumour-size**			
*3–5 cm*	1238 (81.1%)	157 (10.2%)	131 (8.7%)
*5–7 cm*	284 (76.1%)	58 (15.5%)	31 (8.3%)
*>7 cm*	62 (74.7%)	14 (16.9%)	7 (8.4%)
**Surgery**			
*(Bi)Lobar Resection*	1553 (79.7%)	228 (11.7%)	168 (8.6%)
*Sublobar Resection*	31 (94.0%)	1 (3.0%)	1 (3.0%)
**Lymph Node Assessment**			
*Radical Dissection*	1061 (79.1%)	163 (12.2%)	117 (8.7%)
*Sampling*	523 (81.6%)	66 (10.3%)	52 (8.1%)
*Number of nodes dissected (Mean* ± SD)	12.4 ± 6.6	15.7 ± 9.8	15.7 ± 8.6

**Table 3:** Multivariable analysis for unforeseen pN1 and pN2.

N1 Upstaging		
Variable	OR (CI95%)	*p* Value
SUVmax ≥ 6	1.98(1.44–2.73)	<0.0001
Tumour-size ≥ 5 cm	1.52 (1.11–2.10)	0.01
**N2 Upstaging**		
**Variable**	**OR (CI95%)**	***p*** **Value**
Age	0.98 (0.96–0.99)	0.039
Histology (Ref:SCC)	0.48 (0.30–0.78)	0.003
SUVmax ≥ 6	1.807 (1.27–2.58)	0.001
Resected-LN ≥ 6	2.37 (1.26–4.45)	0.007

**Table 4:** An overview of studies on the nodal upstaging.

Study	Patients	Inclusion Criteria	pN1/N2-Upstaging (%)	Upstaging Risk Factors	Survival pN0	Survival u-pN1	Survival u-pN2
**Rocha, 2004 [10]** (prospective; thoracotomy)	109	c-stage: I/II (cN0, cN1) NSCLC	upN1:5.5% upN2: 8.3%	-lower lobe location (*p* < 0.006)	NA	NA	NA
**Lee, 2007 [14]** (retrospective; thoracotomy)	224	c-stage: I NSCLC	upN1: 9.8% upN2:6.5% (T1)-8.7% (T2)	-central tumours (*p* < 0.001) -larger cT size (*p* < 0.001) -adeno-carcinoma histology (*p*: 0.082) -higher tumour PET-SUV_max_ (*p*: 0.017)	NA	NA	NA
**Licht, 2013 [1]** (retrospective on a National registry; Thoracotomy vs. VATS)	1513	c-stage: I NSCLC	upN1: 13.1% vs. 8.1% (*p* < 0.001) upN2: 11.5 vs. 3.8% (*p* < 0.001)	-cT stage (*p* = 0.01) -invasive mediastinal staging (*p* < 0.001) -number of nodal stations dissected (*p* = 0.02) -surgical approach (*p* < 0.001) -lower lobe (*p* = 0.045).	HR: 1	HR:1.84	HR: 2.79 (*p* < 0.001)
**Marulli, 2018 [12]** (retrospective; VATS)	231	cT1-T3N0, I-IIB NSCLC	upN1: 9.1% upN2: 7.4%	-T size (*p*: 0.027) -adenocarcinoma histology (*p*: 0.0382)	NA	NA	NA
**Ismail, 2018 [15]** (retrospective; VATS)	136	c-stage: I-IIB	upN1: 7.4% upN2: 5.2%	-positive nodes in stations 2–4 (0.009) and 5–6 (0.027)	NA	NA	NA
**Moon, 2018 [11]** (retrospective; Thoracotomy)	486	Peripheral cT1N0	upN1: 4.7% upN2: 3.9%	-tumour diameter (*p*: 0.039) -consolidation/tumour ratio (*p* = 0.001)	NA	NA	NA
**Marulli, 2019 [13]** (retrospective on a National registry; VATS)	3276	cT1-T3N0, I-IIB NSCLC	upN1: 6.2% upN2: 2.4%	-adenocarcinoma histology (*p* < 0.001) -higher tumour grade (*p* < 0.001) -higher pathologicT status (*p* < 0.001) -tumour size > 3 cm (*p* < 0.001) -upper lobe tumours (*p* = 0.049) ->12 nodes resected (*p* < 0.001)	NA	NA	NA
**Present series** **2021** (retrospective on a National registry; VATS)	1982	cN0 peripheral solid-type NSCLC > 3 cm	upN1: 11.6% upN2: 8.5%	uN1: -SUVmax (OR: 1.98; CI95: 1.44–2.73, *p* = 0.0001), -tumour-size (OR: 1.52; CI: 1.11–2.10, *p* = 0.01); uN2: -age, *p* = 0.039), -histology (*p* = 0.003), -SUVmax (*p* = 0.015), -number of resected nodes (*p* = 0.002).	5 y: 73%	5 y: 35%	5 y: 31% (*p* < 0.0001)

## Data Availability

Data are the property of the Italian VATS group.
